# Cemented versus Cementless Femoral Fixation for Elective Primary Total Hip Arthroplasty: A Nationwide Analysis of Short-Term Complication and Readmission Rates

**DOI:** 10.3390/jcm12123945

**Published:** 2023-06-09

**Authors:** Xiao T. Chen, Alexander B. Christ, Brian C. Chung, Andy Ton, Alexander M. Ballatori, Shane Shahrestani, Brandon S. Gettleman, Nathanael D. Heckmann

**Affiliations:** 1 Department of Orthopaedic Surgery, Keck School of Medicine, University of Southern California, Los Angeles, CA 91803, USA; tonythechen@gmail.com (X.T.C.); chungbri@usc.edu (B.C.C.); shane_shahrestani@yahoo.com (S.S.); 2 University of South Carolina School of Medicine, Columbia, SC 29209, USA; brandon.gettleman63@gmail.com

**Keywords:** total hip arthroplasty, cemented, cementless, complication, readmission

## Abstract

Cementless fixation during total hip arthroplasty (THA) is the predominant mode of fixation utilized for both acetabular and femoral components during elective primary THAs performed in the United States. This study aims to compare early complication and readmission rates between primary THA patients receiving cemented versus cementless femoral fixation. The 2016–2017 National Readmissions Database was queried to identify patients undergoing elective primary THA. Postoperative complication and readmission rates at 30, 90, and 180 days were compared between cemented and cementless cohorts. Univariate analysis was conducted to compare differences between cohorts. Multivariate analysis was performed to account for confounding variables. Of 447,902 patients, 35,226 (7.9%) received cemented femoral fixation, while 412,676 (92.1%) did not. The cemented group was older (70.0 vs. 64.8, *p* < 0.001), more female (65.0% vs. 54.3%, *p* < 0.001), and more comorbid (CCI 3.65 vs. 3.22, *p* < 0.001) compared to the cementless group. On univariate analysis, the cemented cohort had decreased odds of periprosthetic fracture at 30 days postoperatively (OR: 0.556, 95%-CI 0.424–0.729, *p* < 0.0001), but higher odds of hip dislocation, periprosthetic joint infection, aseptic loosening, wound dehiscence, readmission, medical complications, and death at all timepoints. On multivariate analysis, the cemented fixation cohort demonstrated reduced odds of periprosthetic fracture at all postoperative timepoints: 30 (OR: 0.350, 95%-CI 0.233–0.506, *p* < 0.0001), 90 (OR: 0.544, 95%-CI 0.400–0.725, *p* < 0.0001), and 180 days (OR: 0.573, 95%-CI 0.396–0.803, *p* = 0.002). Cemented femoral fixation was associated with significantly fewer short-term periprosthetic fractures, but more unplanned readmissions, deaths, and postoperative complications compared to cementless femoral fixation in patients undergoing elective THA.

## 1. Introduction

Several million patients worldwide undergo total hip arthroplasty (THA) annually, with over 1.6 million THAs performed among Organization for Economic Cooperation and Development (OECD) countries in 2011 [1]. The annual incidence of THA has dramatically risen over the last decade. In OECD countries, THA rates increased by 30% between 2007–2017 [2]. In the United States, over 456,000 total or partial hip replacements were performed in 2010 [3], while the 2010 prevalence of THA exceeded 2.55 million individuals [4].

The increasing popularity of THA has been accompanied by various implant types and surgical techniques. One topic of ongoing controversy among orthopedic surgeons is the usage of cemented femoral fixation in primary THA. Over 93% of primary THAs within the United States utilize cementless femoral fixation [5,6], yet international registries from Australia, New Zealand, Norway, England, and Sweden demonstrate a preference towards cemented femoral fixation [6]. Previous studies have demonstrated an association between cemented femoral fixation and fewer periprosthetic fractures, lower incidence of thigh pain, decreased odds of revision surgery, versatility in patients with poor bone quality, and superior long-term implant survival in older patients [7,8,9,10]. However, surgeons who prefer cementless femoral fixation argue that cementation is more technically challenging, prolongs operative times, and increases the risk of aseptic loosening in younger, high-demand patients [11]. Support for both modes of femoral fixation are often derived from outdated studies that investigated femoral stems or cementation techniques that have been largely discontinued from mainstream use. Due to the paucity of convincing contemporary data, the usage of cemented femoral fixation in primary elective THA remains highly debated.

Few studies have compared postoperative outcomes or readmissions rates in elective THA patients receiving cemented versus cementless femoral fixation. An observational study by Zimmerman et al. of 271 hip osteoarthritis (OA) patients who underwent primary THA found no statistically significant differences in clinical or functional outcomes between those who received uncemented versus hybrid fixation at 12 months postoperatively [12]. In contrast, studies by Emerson et al. and Corten et al. found superior mid-term (7 years) and long-term (17 years) implant survivorship, respectively, in THA patients receiving cementless femoral fixation [13,14]. In a retrospective cohort study of 4019 patients who underwent cemented versus cementless THA for rheumatoid arthritis (RA), cemented composite-beam stems were found to have consistently higher rates of aseptic loosening than cementless stems, though all-cause revision was comparable at 15 years between groups [15]. Prior studies often report conflicting findings, fail to assess readmissions rates, and lack the statistical power to investigate uncommon complications.

The purpose of this study was to assess individuals who underwent elective THA for common indications such as OA, osteonecrosis, and RA of the hip and compare early complication and readmission rates between patients who received cemented versus cementless femoral fixation. We hypothesize that patients who received cementless femoral fixation will have higher rates of readmission and postoperative complications compared to patients who received cemented femoral fixation.

## 2. Materials and Methods

### 2.1. National Readmission Database

The United States National Readmission Database (NRD) is an all-payer, nationally representative database within the Healthcare Cost and Utilization Project that contains data from approximately 18 million inpatient and readmissions records annually. Diagnoses and procedures are coded within each patient admission or readmission using International Classification of Disease, 10th Revision (ICD-10) codes representing data elements such as complications, costs, length of stay, and readmission rates within each calendar year. Data from NRD years before 2016 were excluded, as ICD-9 codes lack the granularity required for this study. This study is exempt from institutional review board approval given that all patient data within the NRD is deidentified.

### 2.2. Patient Selection

A retrospective cohort study was conducted on patients within the 2016–2017 NRD who underwent primary elective THA (Table 1). All patients were identified using ICD-10 codes, and patients with diagnosis codes for OA, RA, osteonecrosis of the hip, and hip dysplasia who underwent subsequent cemented or cementless THA were included for analysis. Exclusion criteria were malignancy, septic arthritis of the hip, abnormal weight loss, anorexia nervosa, malnourishment, fracture of the femur, and history of ipsilateral total or partial hip arthroplasty (Table S1). Demographic and patient information was extracted for the respective cemented and cementless THA cohorts, including age, sex, Charlson comorbidity index (CCI), insurance type, hospital type, income quartile, and discharge location. Unplanned readmission and complications at 30, 90, and 180 days within both cohorts were the primary outcomes of this study. Complications of interest included hip dislocation, periprosthetic joint infection (PJI), periprosthetic fracture, aseptic loosening, wound dehiscence, embolism, thrombosis, hemorrhage, hematoma, seroma, death, and medical complications (acute myocardial infarction (MI), pneumonia, respiratory failure, acute renal failure, cerebral infarction, peripheral nerve injury (PNI), postoperative ileus, urinary tract infection (UTI)) (Table S2). The NRD does not track patients past each calendar year, so patients lacking follow-up at each time point were excluded from analysis at that specific time point.

### 2.3. Statistical Analysis

Patient characteristics, preoperative diagnoses, and hospital characteristics were compared between the two cohorts using standard parametric tests. Chi-squared tests were used to compare categorical variables and student’s *t*-tests were used to compare continuous variables. Multivariate logistic regression analysis was performed with cemented femoral fixation as the independent variable and cementless femoral fixation as the reference. Age, sex, CCI, preoperative diagnosis, insurance type, median income, hospital type, and discharge location were set as dependent variables. Outcomes analyzed on multivariate analysis included dislocation, PJI, periprosthetic fracture, aseptic loosening, wound dehiscence, postoperative hematoma, postoperative seroma, combined medical complications, readmissions, and death at 30, 90, and 180 days postoperatively. All statistical analyses were performed using R studio (RStudio, Boston, MA, USA). A *p*-value < 0.05 was considered statistically significant.

## 3. Results

A total of 884,184 patients who underwent elective THA were initially queried and screened for inclusion. After exclusion criteria were applied, 713,116 patients remained. Of these, 447,902 had ICD-10 codes specifying for cemented or cementless THA. Cemented femoral fixation was performed in 35,226 patients (7.86%), whereas cementless femoral fixation was performed in 412,676 patients (92.14%). The cemented cohort was significantly older (70.0 years vs. 64.8 years, *p* < 0.001), and had more comorbidities (CCI: 3.65 vs. 3.22, *p* < 0.001) and females (65.0% vs. 54.3%, *p* < 0.001) compared to the cementless group. (Table 1).

### 3.1. Readmissions

On univariate analysis, readmission rates were significantly higher in the cemented group at 30 (5.26% vs. 3.43%; *p* < 0.0001), 90 (11.4% vs. 7.79%; *p* < 0.0001), and 180 days (18.3% vs. 13.5%; *p* < 0.0001). (Table 2). 

**Figure 1:**
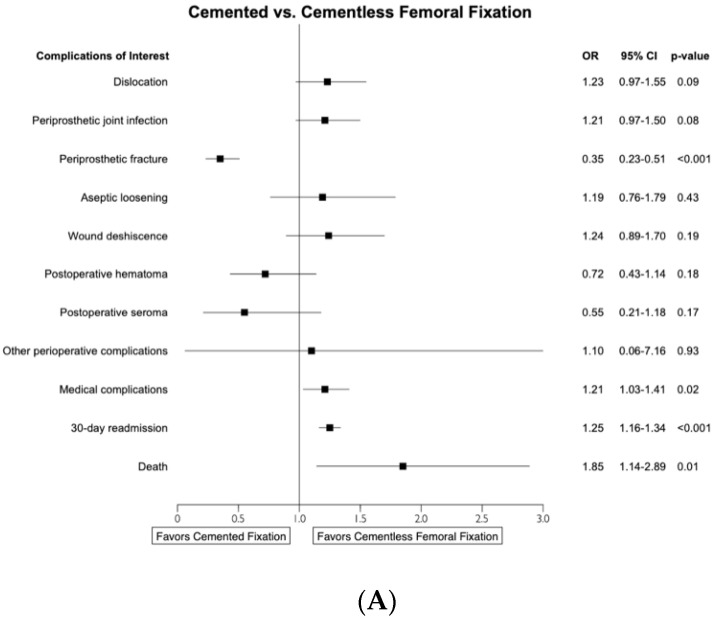

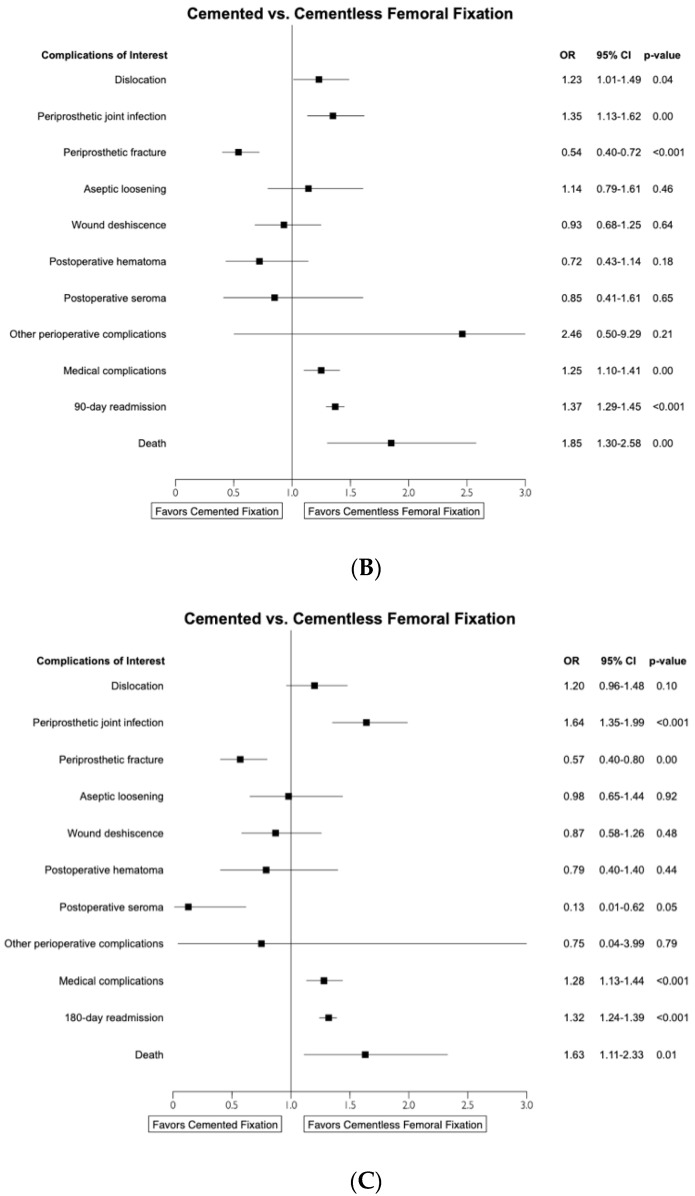
Forest plots of multivariate analysis comparing complication and readmission rates at (**A**) 30 days, (**B**) 90 days, and (**C**) 180 days postoperatively.

After accounting for confounders, multivariate analysis continued to demonstrate higher readmission rates in the cemented cohort at 30 (OR: 1.246, 95%-CI 1.157–1.341, *p* < 0.0001), 90 (OR: 1.371, 95%-CI 1.295–1.451, *p* < 0.0001), and 180 days (OR: 1.316, 95%-CI 1.243–1.392, *p* < 0.0001) postoperatively. (Figure 1A–C, Table 3).

### 3.2. Medical Complications

Complication rates within 30, 90, and 180 days postoperatively were assessed for both the cemented and cementless THA cohorts. At all three postoperative timepoints, patients who underwent cemented femoral fixation had significantly greater odds of acute MI, pneumonia, respiratory failure, acute renal failure, cerebral infarction, UTI, death, and any medical complication (all *p*-values < 0.001). (Table 2) At 90 days postoperatively, the cemented femoral fixation cohort also had significantly higher odds of PNI (OR: 2.018, 95%-CI 1.063–3.832, *p* = 0.048) and postoperative ileus (OR: 1.897, 95%-CI 1.339–2.687, *p* = 0.0002). (Table 2B) Multivariate analysis continued to show that patients who underwent cemented femoral fixation were at greater odds of all medical complications (30 days: OR: 1.209, 95%-CI 1.034–1.411, *p* = 0.0169; 90 days: OR: 1.25, 95%-CI 1.105–1.413, *p* = 0.0004; 180 days: OR: 1.280, 95%-CI 1.132–1.444, *p* < 0.0001) and death (30 days: OR: 1.854, 95%-CI 1.135–2.892, *p* = 0.0093; 90 days: OR: 1.852, 95%-CI 1.302–2.575, *p* = 0.00039; 180 days: OR: 1.633, 95%-CI 1.111–2.327, *p* = 0.0091) at all three timepoints. (Figure 1A–C, Table 3).

### 3.3. Surgical Complications

On univariate analysis, the cemented cohort demonstrated reduced odds of periprosthetic fracture at 30 days (OR: 0.556, 95%-CI 0.424 to 0.729, *p* < 0.0001), but not at 90 (OR: 0.875, 95%-CI 0.707 to 1.08, *p* = 0.221) or 180 days (OR: 0.919, 95%-CI 0.715 to 1.182, *p* = 0.510). However, at 30, 90, and 180 days postoperatively, patients with cemented THAs were also more likely to experience hip dislocation, PJI, aseptic loosening, and wound dehiscence (all *p*-values < 0.001) (Table 2). After accounting for confounding factors, the cemented fixation cohort demonstrated reduced odds of periprosthetic fracture at all postoperative time points: 30 (OR: 0.350, 95%-CI 0.233–0.506, *p* < 0.0001), 90 (OR: 0.544, 95%-CI 0.400–0.725, *p* < 0.0001), and 180 days (OR: 0.573, 95%-CI 0.396–0.803, *p* = 0.002). (Figure 1A–C, Table 3) No other surgical complications were found to be significantly different between the two cohorts at 30 days postoperatively. However, cemented THAs had greater odds of hip dislocation at 90 days (OR: 1.232, 95%-CI 1.011–1.492, *p* = 0.036) and PJI at 90 (OR: 1.354, 95%-CI 1.128–1.618, *p* = 0.001) and 180 days (OR: 1.644, 95%-CI 1.349–1.992, *p* < 0.0001) (Figure 1B,C, Table 3).

## 4. Discussion

The present study found a decreased risk of periprosthetic fracture, but increased risk of readmission, death, and postoperative complications for elective THA patients receiving cemented femoral fixation compared to cementless femoral fixation. Elective THA patients who received cemented femoral fixation were older, more comorbid, and more likely to be female compared to those who received cementless THAs. Univariate analysis demonstrated significantly lower odds of periprosthetic fracture at 30 days postoperatively but not at 90 or 180 days in the cemented THA cohort. These same patients had greater odds of readmission, hip dislocation, PJI, aseptic loosening, medical complications, and death at all three time points. However, after accounting for confounding factors, patients undergoing cemented femoral fixation were found to have significantly reduced odds of periprosthetic fracture at 30, 90 and 180 days, though they continued to exhibit greater odds of readmission, death, and medical complications at all time points.

Unplanned hospital readmission following primary THA has become an important quality-of-care metric over the past decade with the introduction of the 2012 U.S. Center for Medicare & Medicaid (CMS) Bundled Payments for Care Improvement (BPCI) initiative, 2012 CMS Hospital Readmissions Reduction Program (HRRP), and 2016 Comprehensive Care for Joint Replacement (CJR) reimbursement model. These policies have been shifting reimbursement from the traditional fee-for-service model towards bundled payments, [16] with multiple studies demonstrating that unplanned readmissions in total joint arthroplasty cases are associated with substantially increased costs of care and major reductions in hospital reimbursement [17,18,19,20,21,22]. The BCPI utilizes a 90-day bundle, the HRRP uses a 30-day benchmark, and the CJR has both 30- and 90-day metrics, making 30 and 90 days critical postoperative timepoints. This study found similar 30-day readmission rates for patients who underwent cementless THAs (3.43%) to those reported by Mednick et al. [23] (3.65%) and Paxton et al. [24] (3.6%). Additionally, the pooled readmissions rate of both cohorts at 90 days (8.0%) was similar to that of Ramkumar et al., who found a 7.7% unplanned readmissions rate following primary elective unilateral THAs [25]. In the present study, readmission rates were significantly higher at all three time points in the cemented femoral fixation cohort on both univariate and multivariate analysis. However, cemented femoral fixation itself is unlikely to be the root cause of increased short-term readmission rates, and there are likely uncontrolled confounders within the older and more comorbid cemented THA cohort. Future studies are needed to further parse out the exact risk factors for readmission in this particular subset of THA patients.

The incidence of postoperative surgical complications varied enormously between cohorts in this study, with advantages and disadvantages for each type of fixation. Patients who received cementless THAs were 1.8–2.9 times more likely to experience a periprosthetic fracture on multivariate analysis, a finding that has been well-described in prior literature. Berry found a periprosthetic fracture rate of 5.4% in cementless THAs (versus 0.3% in cemented THAs) within the Mayo Clinic Total Joint Registry, while Springer et al. used the American Joint Replacement Registry and reported a 2.6-times higher periprosthetic fracture rate within 90 days postoperatively in THA patients who received cementless femoral stems versus cemented femoral stems [26,27]. However, THA patients in this study who underwent cemented femoral fixation were 1.2–1.6 times more likely to experience PJI, hip dislocation, or aseptic loosening compared to THA patients who received cementless femoral stems. These findings mirror those of Yoon et al., which found 1.53-times increased odds of PJI in cemented THAs compared to cementless [28].

It is unlikely that cementation itself is a risk for PJI, and differences are more likely related to selection bias as many surgeons in the United States preferentially utilize cemented fixation in frail, elderly, sicker patients. The increased PJI that persisted in our multivariate analysis is likely the result of unidentified confounders contributing to this finding, considering the large comorbid burden of the cemented THA cohort. Prior studies have also reported greater rates of osteolysis and aseptic loosening in cemented femoral stems, often leading to revision surgery [29,30]. Kurtz et al. found that 59% of total readmission costs following THA within 90 days were associated with complications requiring operative management such as PJI, hip dislocation, and periprosthetic fractures [31]. Further prospective studies are needed to account for surgeon selection bias in order to account for both identifiable and unidentifiable confounders.

This study also found cemented femoral fixation to be associated with increased rates of dislocation at 90 days postoperatively on multivariate analysis. However, this group was also older, sicker, and had more female patients than the cementless group. Female gender, increasing age, and conditions associated with increasing age, such as spinal fusion and Parkinson’s disease, have been shown to increase the risk of dislocation [32]. Although several of these variables are unaccounted for in the Charlson comorbidity index, the overall effects of surgeon selection bias choosing cemented fixation in more frail patients may not be fully captured by the CCI. Additionally, it can be hypothesized that by using cemented femoral fixation, surgeons are identifying patients with osteopenia and other comorbid conditions. These patients may be at higher risk for any number of complications, and their degree of frailty may not be adequately captured by an administrative coding database.

This study has several limitations. First, the use of retrospective data from the NRD in this study introduces selection bias related to surgeon preference for cementing femoral components in patients who are older, more comorbid, and have poorer bone stock. This is particularly true using a United States nationwide sample, given the widespread utilization of cementless femoral fixation as the preferred mode of femoral fixation. However, we attempted to mitigate these confounding factors by using a multivariate model accounting for age, sex, and comorbidity burden. Despite our multivariate model, there are likely unaccounted-for confounders driving the increased risk of postoperative complications observed in the cemented cohort. Second, as with any administrative database study, the quality of our data depends on the accuracy of ICD-10 coding. However, we do not think that these inaccuracies affected our results, as there is no evidence that these types of inaccuracies would be more prevalent in one of the two comparative cohorts in this study. Third, the NRD aggregates data from 28 states, representing approximately 60% of the US population, which may limit the generalizability of findings from this study to specific geographic regions. Fourth, the NRD does not compile patient-reported outcomes such as pre- and postoperative pain and function, intraoperative variables such as blood loss and operative time, preoperative radiographic findings, or surgeon characteristics such as years of experience, fixation preference, and surgical volume. Fifth, our study only analyzed patients between 2016–2017, with no follow-up past 1 year. However, this time frame was chosen because the ICD-10 codes implemented at the end of 2015 specified the mode of femoral fixation, whereas the prior ICD-9 codes lacked such granularity. Sixth, multiple preoperative diagnoses were permitted for the elective THAs queried in this study. This decision was in accordance with the majority of prior randomized controlled trials [10,33,34] that included elective THAs for multiple different diagnoses, so the results in this study may be more broadly generalizable but not specific to any particular pathology. Finally, this study was unable to account for implant type, cementation technique, and surgical approach. Factors such as implant geometry, materials, surface finishes, and bearings may have introduced heterogeneity into our findings. While we acknowledge our inability to identify specific implants is a weakness of our study, we do not believe it diminishes our findings in any meaningful way.

To our knowledge, this is the first and largest national database study to analyze the relationship between femoral fixation type, unplanned readmissions, and postoperative complications following primary elective THA. The usage of new ICD-10 codes allowed for a granular and contemporary analysis of femoral fixation that was not previously possible using ICD-9 documentation. The novel findings within this study provide orthopedic surgeons with additional information regarding the different risks associated with each type of femoral fixation in primary elective THA patients. Future studies are needed to determine long-term outcomes and appropriate indications for each fixation type.

## 5. Conclusions

In conclusion, this study used a large, nationally representative database to assess postoperative complication and readmission rates in patients undergoing primary elective THA with either cemented or cementless femoral fixation. After accounting for confounding factors, THA patients who received cemented femoral stems demonstrated significantly lower odds of periprosthetic fracture, but greater incidence of unplanned readmission, PJI, hip dislocation, aseptic loosening, death, and medical complications. Arthroplasty surgeons should consider these findings in conjunction with their clinical judgment when deciding on the ideal method of femoral fixation for primary THAs.

## Figures and Tables

**Table 1:** Patient information and surgical indications.

	Cemented Total Hip Arthroplasty Patients (*n* = 35,226)	Cementless Total Hip Arthroplasty Patients (*n* = 412,676)	*p*-Value
**Age (years), mean ± SD**	69.97 ± 12.39	64.81 ± 11.22	***p* < 0.0001**
**Sex**			
Female, *n* (%)	22,890 (65.0%)	224,083 (54.3%)	***p* < 0.0001**
Male, *n* (%)	12,336 (35.0%)	188,593 (45.7%)	
**Charlson Comorbidity Index Score, mean ± SD**	3.65 ± 1.29	3.22 ± 1.26	
History of myocardial infarction	3107 (8.82%)	29,699 (7.20%)	***p* < 0.0001**
Congestive heart failure	1300 (3.69%)	9275 (2.25%)	
Peripheral vascular disease	1520 (4.31%)	16,443 (3.98%)	
History of a cerebrovascular accident or transient ischemic attack	1778 (5.05%)	20,749 (5.03%)	
Dementia	0 (0%)	0 (0%)	
Chronic obstructive pulmonary disease	1048 (2.98%)	14,848 (3.60%)	
Connective tissue disease	244 (0.69%)	1824 (0.44%)	
Peptic ulcer disease	0 (0%)	0 (0%)	
Liver disease	16 (0.05%)	135 (0.03%)	
Diabetes mellitus	3994 11.3(%)	46,285 (11.2%)	
Hemiplegia	118 (0.34%)	1406 (0.34%)	
Moderate to severe chronic kidney disease	1212 (%)	6797 (1.65%)	
Solid tumor	0 (0%)	0 (0%)	
Leukemia/Lymphoma	239 (3.44%)	1005 (0.24%)	
Acquired immunodeficiency syndrome	34 (0.10%)	421 (0.10%)	
**Surgical Indication**			
Primary osteoarthritis of the hip	25,878 (73.5%)	358,200 (86.8%)	***p* < 0.0001**
Rheumatoid arthritis of the hip	13 (0.04%)	54 (0.01%)	
Osteonecrosis of the hip	1004 (2.85%)	10,802 (2.62%)	
Secondary osteoarthritis of the hip	683 (1.94%)	6255 (1.52%)	
Hip dysplasia	177 (0.50%)	2546 (0.62%)	
Other	7474 (21.2%)	34,819 (8.44%)	
**Insurance**			
Medicare, *n* (%)	24,206 (68.7%)	218,208 (52.9%)	***p* < 0.0001**
Medicaid, *n* (%)	1515 (4.30%)	19,232 (4.66%)	
Private, *n* (%)	8516 (24.2%)	163,161 (39.5%)	
Other (include self-pay, no charge, and other), *n* (%)	955 (2.71%)	11,737 (2.84%)	
**Median income by zip code**			
Quartile 1, *n* (%)	6927 (19.7%)	79,423 (19.3%)	***p* < 0.0001**
Quartile 2, *n* (%)	9786 (27.8%)	108,169 (26.2%)	
Quartile 3, *n* (%)	9426 (26.8%)	114,520 (27.8%)	
Quartile 4, *n* (%)	8661 (24.6%)	104,993 (25.4%)	
**Hospital type**			
Metropolitan non-teaching, *n* (%)	8788 (24.9%)	115,905 (28.1%)	***p* < 0.0001**
Metropolitan teaching, *n* (%)	22,966 (65.2%)	257,168 (62.3%)	
Non-metropolitan, *n* (%)	3472 (9.9%)	39,603 (9.60%)	
**Discharge location**			
Routine/Home, *n* (%)	10,703 (30.4%)	172,556 (41.8%)	***p* < 0.0001**
Short-term hospital, *n* (%)	114 (0.33%)	499 (0.12%)	
Skilled nursing facility, *n* (%)	10,869 (30.9%)	58,864 (14.3%)	
Home health care, *n* (%)	13,413 (38.1%)	180,165 (43.7%)	

**Table 2:** (A) Univariate analysis comparing complication and readmission rates at 30 days postoperatively. (B) Univariate analysis comparing complication and readmission rates at 90 days postoperatively. (C) Univariate analysis comparing complication and readmission rates at 180 days postoperatively.

(A)					
Complications within 30 Days	Cemented Total Hip Arthroplasty Patients (*n* = 32,507)	Cementless Total Hip Arthroplasty Patients (*n* = 375,658)	OR	95% CI	*p*-Value
Readmission for any reason	1709 (5.26%)	12,894 (3.43%)	**2.558**	**2.450 to 2.670**	**<0.0001**
Dislocation of internal hip prosthesis	198 (0.61%)	1050 (0.28%)	**2.186**	**1.878 to 2.546**	**<0.0001**
Periprosthetic fracture	55 (0.17%)	1141 (0.30%)	**0.5563**	**0.4243 to 0.7293**	**<0.0001**
Periprosthetic joint infection	227 (0.70%)	1479 (0.39%)	**1.779**	**1.546 to 2.047**	**<0.0001**
Aseptic loosening	69 (0.21%)	311 (0.08%)	**2.567**	**1.977 to 3.333**	**<0.0001**
Embolism due to internal orthopedic prosthetic devices, implants and grafts	6 (0.02%)	26 (0.007%)	**2.667**	**1.098 to 6.480**	***p* = 0.0383**
Thrombosis due to internal orthopedic prosthetic devices, implants and grafts	0 (0%)	8 (0.002%)	0.6798	0.03923 to 11.78	>0.9999
Wound injury	93 (0.29%)	579 (0.15%)	**1.859**	**1.493 to 2.314**	**<0.0001**
Postprocedural hemorrhage	45 (0.14%)	411 (0.11%)	1.266	0.9302 to 1.722	0.1329
Postprocedural hematoma	36 (0.11%)	382 (0.10%)	1.089	0.7738 to 1.533	0.6243
Postprocedural seroma	11 (0.03%)	163 (0.04%)	0.7798	0.4234 to 1.436	0.486
Other intraoperative and postprocedural complications	6 (0.02%)	11 (0.003%)	**6.304**	**2.331 to 17.05**	**0.0015**
Acute myocardial infarction	67 (0.21%)	340 (0.09%)	**2.28**	**1.754 to 2.963**	**<0.0001**
Pneumonia	143 (0.44%)	719 (0.19%)	**2.304**	**1.925 to 2.758**	**<0.0001**
Respiratory failure	186 (0.57%)	1021 (0.27%)	**2.112**	**1.805 to 2.470**	**<0.0001**
Acute renal failure	250 (0.77%)	1480 (0.39%)	**1.959**	**1.713 to 2.242**	**<0.0001**
Cerebral infarction	34 (0.10%)	125 (0.03%)	**3.146**	**2.153 to 4.596**	**<0.0001**
Peripheral nerve injury	2 (0.006%)	41 (0.01%)	0.5637	0.1363 to 2.331	0.5794
Death	40 (0.12%)	126 (0.03%)	**3.672**	**2.572 to 5.241**	**<0.0001**
Postoperative ileus	18 (0.06%)	188 (0.05%)	1.107	0.6822 to 1.795	0.6984
Urinary tract infection	147 (0.45%)	1052 (0.28%)	**1.618**	**1.361 to 1.923**	**<0.0001**
All medical complications	887 (2.73%)	5092 (1.36%)	**2.041**	**1.899 to 2.194**	**<0.0001**
**(B)**					
**Complications within 90 days**	**Cemented Total Hip Arthroplasty Patients (*n* = 26,641)**	**Cementless Total Hip Arthroplasty Patients (*n* = 302,971)**	**OR**	**95% CI**	***p*** **-Value**
Readmission for any reason	3038 (11.4%)	23,587 (7.79%)	**1.525**	**1.465 to 1.587**	**<0.0001**
Dislocation of internal hip prosthesis	284 (1.07%)	1501 (0.50%)	**2.164**	**1.905 to 2.458**	**<0.0001**
Periprosthetic fracture	91 (0.34%)	1182 (0.39%)	**0.8751**	**0.7068 to 1.083**	**0.2206**
Periprosthetic joint infection	335 (1.26%)	1815 (0.60%)	**2.113**	**1.879 to 2.376**	**<0.0001**
Aseptic loosening	92 (0.35%)	446 (0.15%)	**2.351**	**1.877 to 2.943**	**<0.0001**
Embolism due to internal orthopedic prosthetic devices, implants and grafts	6 (0.02%)	32 (0.01%)	2.133	0.8916 to 5.101	0.1232
Thrombosis due to internal orthopedic prosthetic devices, implants and grafts	0 (0%)	9 (0.003%)	0.5985	0.03483 to 10.28	>0.9999
Wound injury	96 (0.36%)	739 (0.24%)	**1.479**	**1.195 to 1.830**	**0.0003**
Postprocedural hemorrhage	48 (0.18%)	466 (0.15%)	1.172	0.8703 to 1.577	0.2958
Postprocedural hematoma	40 (0.15%)	335 (0.11%)	1.358	0.9784 to 1.886	0.0662
Postprocedural seroma	20 (0.08%)	152 (0.05%)	1.497	0.9389 to 2.386	0.0925
Other intraoperative and postprocedural complications	9 (0.03%)	14 (0.005%)	**7.313**	**3.165 to 16.90**	**<0.0001**
Acute myocardial infarction	81 (0.30%)	460 (0.15%)	**2.006**	**1.583 to 2.541**	**<0.0001**
Pneumonia	210 (0.79%)	1002 (0.33%)	**2.394**	**2.062 to 2.780**	**<0.0001**
Respiratory failure	291 (1.09%)	1454 (0.48%)	**2.29**	**2.018 to 2.599**	**<0.0001**
Acute renal failure	419 (1.57%)	2103 (0.69%)	**2.286**	**2.057 to 2.541**	**<0.0001**
Cerebral infarction	47 (0.18%)	226 (0.07%)	**2.367**	**1.729 to 3.242**	**<0.0001**
Peripheral nerve injury	11 (0.04%)	62 (0.02%)	**2.018**	**1.063 to 3.832**	**0.0479**
Death	76 (0.29%)	245 (0.08%)	**3.535**	**2.732 to 4.574**	**<0.0001**
Postoperative ileus	37 (0.14%)	222 (0.07%)	**1.897**	**1.339 to 2.687**	**0.0002**
Urinary tract infection	276 (1.04%)	1449 (0.48%)	**2.178**	**1.914 to 2.479**	**<0.0001**
All medical complications	1448 (5.44%)	7223 (2.38%)	**2.353**	**2.221 to 2.494**	**<0.0001**
**(C)**					
**Complications within 180 days**	**Cemented Total Hip Arthroplasty Patients (*n* = 17,970)**	**Cementless Total Hip Arthroplasty Patients (*n* = 203,987)**	**OR**	**95% CI**	***p*** **-Value**
Readmission for any reason	3286 (18.3%)	27,506 (13.5%)	**1.436**	**1.380 to 1.494**	**<0.0001**
Dislocation of internal hip prosthesis	205 (1.14%)	1252 (0.61%)	**1.869**	**1.611 to 2.168**	**<0.0001**
Periprosthetic fracture	66 (0.37%)	815 (0.40%)	0.919	0.7148 to 1.182	0.5097
Periprosthetic joint infection	271 (1.51%)	1342 (0.66%)	**2.312**	**2.027 to 2.637**	**<0.0001**
Aseptic loosening	66 (0.37%)	402 (0.20%)	**1.867**	**1.438 to 2.423**	**<0.0001**
Embolism due to internal orthopedic prosthetic devices, implants and grafts	3 (0.02%)	19 (0.009%)	1.792	0.5304 to 6.058	0.4177
Thrombosis due to internal orthopedic prosthetic devices, implants and grafts	0 (0%)	7 (0.003%)	0.7567	0.04322 to 13.25	>0.9999
Wound injury	62 (0.35%)	499 (0.24%)	**1.412**	**1.084 to 1.839**	**0.0102**
Postprocedural hemorrhage	36 (0.20%)	335 (0.16%)	1.22	0.8650 to 1.722	0.256
Postprocedural hematoma	20 (0.11%)	204 (0.10%)	1.113	0.7030 to 1.762	0.6235
Postprocedural seroma	2 (0.01%)	99 (0.05%)	**0.2292**	**0.05653 to 0.9295**	**0.0169**
Other intraoperative and postprocedural complications	1 (0.006%)	26 (0.01%)	0.4366	0.05924 to 3.217	0.7212
Acute myocardial infarction	68 (0.38%)	494 (0.24%)	**1.565**	**1.214 to 2.017**	**0.0005**
Pneumonia	224 (1.25%)	953 (0.47%)	**2.689**	**2.323 to 3.113**	**<0.0001**
Respiratory failure	280 (1.56%)	1419 (0.70%)	**2.26**	**1.986 to 2.571**	**<0.0001**
Acute renal failure	410 (2.28%)	2044 (1.00%)	**2.307**	**2.072 to 2.568**	**<0.0001**
Cerebral infarction	55 (0.31%)	241 (0.12%)	**2.595**	**1.936 to 3.480**	**<0.0001**
Peripheral nerve injury	9 (0.05%)	82 (0.04%)	1.246	0.6260 to 2.480	0.5609
Death	64 (0.36%)	230 (0.11%)	**3.166**	**2.399 to 4.179**	**<0.0001**
Postoperative ileus	26 (0.14%)	199 (0.10%)	1.484	0.9857 to 2.234	0.057
Urinary tract infection	274 (1.52%)	1457 (0.71%)	**2.152**	**1.890 to 2.451**	**<0.0001**
All medical complications	1410 (7.85%)	7119 (3.49%)	**2.355**	**2.219 to 2.498**	**<0.0001**

**Table 3:** Multivariate analysis comparing complication and readmission rates at 30 days, 90 days, and 180 days postoperatively. Cemented femoral fixation was the independent variable while cementless femoral fixation was set as the reference.

30-Day Outcomes	Adjusted OR	95% CI	*p*-Value
Dislocation	1.2279	0.9677–1.5449	0.08511
Periprosthetic joint infection	1.2121	0.9733–1.4990	0.080586
Periprosthetic fracture	**0.3498**	**0.2326–0.5058**	**<0.0001**
Aseptic loosening	1.1871	0.7601–1.7871	0.429851
Wound dehiscence	1.2426	0.8910–1.6996	0.18651
Postoperative hematoma	0.7164	0.4262–1.1372	0.18051
Postoperative seroma	0.5493	0.2107–1.1800	0.16546
Other perioperative complications	1.1009	0.0558–7.1626	0.9317
30-day readmission	**1.2462**	**1.1569–1.3408**	**<0.0001**
Medical complications	**1.2086**	**1.0338–1.4109**	**0.01689**
Death	**1.8541**	**1.1353–2.8919**	**0.0093**
**90-day outcomes**	**Adjusted OR**	**95% CI**	***p*-value**
Dislocation	**1.2322**	**1.0111–1.4924**	**0.035461**
Periprosthetic joint infection	**1.354**	**1.1277–1.6176**	**0.000985**
Periprosthetic fracture	**0.5441**	**0.4000–0.7248**	**<0.0001**
Aseptic loosening	1.1431	0.7901–1.6122	0.46126
Wound dehiscence	0.9289	0.6751–1.2532	0.639764
Postoperative hematoma	0.7212	0.4335–1.1372	0.18177
Postoperative seroma	0.8527	0.4064–1.6076	0.6464
Other perioperative complications	2.455	0.5028–9.2865	0.2127
90-day readmission	**1.3711**	**1.2948–1.4510**	**<0.0001**
Medical complications	**1.25**	**1.1045–1.4131**	**0.000383**
Death	**1.8523**	**1.3015–2.5750**	**0.000385**
**180-day outcomes**	**Adjusted OR**	**95% CI**	***p*-value**
Dislocation	1.2004	0.9622–1.4849	0.098667
Periprosthetic joint infection	**1.6442**	**1.3491–1.9921**	**<0.0001**
Periprosthetic fracture	**0.5725**	**0.3959–0.8031**	**0.00194**
Aseptic loosening	0.9796	0.6461–1.4351	0.91915
Wound dehiscence	0.8684	0.5785–1.2603	0.47622
Postoperative hematoma	0.7865	0.4044–1.3965	0.44353
Postoperative seroma	**0.1346**	**0.0076–0.6236**	**0.0482**
Other perioperative complications	0.7502	0.0405–3.9929	0.7859
180-day readmission	**1.3156**	**1.2428–1.3920**	**<0.0001**
Medical complications	**1.2796**	**1.1322–1.4443**	**<0.0001**
Death	**1.6333**	**1.1112–2.3273**	**0.009075**

## Data Availability

Data for this study can be found on the National Registration Database.

## Supplementary Materials

The following supporting information can be downloaded at: https://www.mdpi.com/article/10.3390/jcm12123945/s1, Table S1: Procedure, diagnosis, and exclusion codes. Table S2. Complication Codes.
