# Supplementary material for: Cemented versus Cementless Femoral Fixation for Elective Primary Total Hip Arthroplasty: A Nationwide Analysis of Short-Term Complication and Readmission Rates
Source: J Clin Med. 2023 Jun 9;12(12):3945. doi: 10.3390/jcm12123945 (PMC10299501; doi:10.3390/jcm12123945)
Supplement: Supplementary file 1 [file jcm-12-03945-s001.zip › jcm-2442332-supplementary.pdf]

Supplementary Materials:

Table S1: Procedure, diagnosis, and exclusion codes

**Procedure**	**ICD-10 Codes**
All total hip arthroplasty	“0SRB019”, “0SR9019”, “0SRB029”, “0SR9029”, “0SRB039”, “0SR9039”, “0SRB049”, “0SR9049”, “0SR9069”, “0SR90J9”, “0SRB069”, “0SRB0J9”, “0SRB01A”, “0SR901A”, “0SRB02A”, “0SR902A”, “0SRB03A”, “0SR903A”, “0SRB04A”, “0SR904A”, “0SR906A”, “0SR90JA”, “0SRB06A”, “0SRB0JA”, “0SR901Z”, “0SR902Z”, “0SR903Z”, “0SR904Z”, “0SR906Z”, “0SR907Z”, “0SR90EZ”, “0SR90JZ”, “0SR90KZ”, “0SRB01Z”, “0SRB02Z”, “0SRB03Z”, “0SRB04Z”, “0SRB06Z”, “0SRB07Z”, “0SRB0EZ”, “0SRB0JZ”, “0SRB0KZ”
Cemented total hip arthroplasty	“0SRB019”, “0SR9019”, “0SRB029”, “0SR9029”, “0SRB039”, “0SR9039”, “0SRB049”, “0SR9049”, “0SR9069”, “0SR90J9”, “0SRB069”, “0SRB0J9”
Cementless total hip arthroplasty	“0SRB01A”, “0SR901A”, “0SRB02A”, “0SR902A”, “0SRB03A”, “0SR903A”, “0SRB04A”, “0SR904A”, “0SR906A”, “0SR90JA”, “0SRB06A”, “0SRB0JA”
**Diagnosis**	
Primary osteoarthritis of the hip	“M1611”, “M1612”, “M160”, “M1610”
Rheumatoid arthritis of the hip	“M05151”, “M05152”, “M05159”, “M05251”, “M05252”, “M05259”, “M05351”, “M05352”, “M05359”, “M05451”, “M05452”, “M05459”, “M05551”, “M05552”, “M05559”, “M05651”, “M05652”, “M05659”, “M05751”, “M05752”, “M05759”, “M05851”, “M05852”, “M05859”, “M05051”, “M05052”, “M05059”
Osteonecrosis of the hip	“M879”, “M87350”, “M87351”, “M87352”,“M87353”, “M87850”, “M87851”, “M87852”, “M87859”, “M87050”, “M87051”, “M87052”, “M87059”, “M87150”, “M87151”, “M87152”, “M87159”, “M87250”, “M87251”, “M87252”, “M87256”
Secondary osteoarthritis of the hip	“M1652”, “M1651”, “M1650”, “M166”, “M167”
Hip dysplasia	“M162”, “M1630”, “M1631”, “M1632”
**Exclusion Diagnoses/Procedures**	
Abnormal weight loss	“R634”
Anorexia nervosa	“F5000”, “F5001”, “F5002”
Malnourishment	“R627”, “E43”, “E440”, “E441”, “E46”
Malignancy	“C4020”, “C4021”, “C4022”, “C4030”, “C4031”, “C4032”, “C4080”, “C4081”, “C4082”, “C4090”, “C4091”, “C4092, “C414”, “C419”, “C763”, “C7650”, “C7651”, “C7652”, “C96Z”, “C969”
Septic arthritis of the hip	“M00051”, “M00052”, “M00059”, “M00151”, “M00152”, “M00159”, “M00251”, “M00252”, “M00259”, “M00851”, “M00852”, “M00859”, “M01X51”, “M01X52”, “M01X59”

Fracture of femur	“S72001A”, “S72002A”, “S72009A”, “S72011A”, “S72012A”, “S72019A”, “S72021A”, “S72022A”, “S72023A”, “S72024A”, “S72025A”, “S72026A”, “S72031A”, “S72032A”, “S72033A”, “S72034A”, “S72035A”, “S72036A”, “S72041A”, “S72042A”, “S72043A”, “S72044A”, “S72045A”, “S72046A”, “S72051A”, “S72052A”, “S72059A”, “S72061A”, “S72062A”, “S72063A”, “S72064A”, “S72065A”, “S72066A”, “S72091A”, “S72092A”, “S72099A”, “S72101A”, “S72102A”, “S72109A”, “S72111A”, “S72112A”, “S72113A”, “S72114A”, “S72115A”, “S72116A”, “S72121A”, “S72122A”, “S72123A”, “S72124A”, “S72125A”, “S72126A”, “S72131A”, “S72132A”, “S72133A”, “S72134A”, “S72135A”, “S72136A”,“S72141A”, “S72142A”, “S72143A”, “S72144A”, “S72145A”, “S72146A”, "S72001B", "S72001C", "S72002B", "S72002C", "S72009B", "S72009C", "S72011B", "S72011C", "S72012B", "S72012C", "S72019B", "S72019C", "S72021B", "S72021C", "S72022B", "S72022C", "S72023B", "S72023C", "S72024B", "S72024C", "S72025B", "S72025C", "S72026B", "S72026C", "S72031B", "S72031C", "S72032B", "S72032C", "S72033B", "S72033C", "S72034B", "S72034C", "S72034B", "S72034C", "S72035B", "S72035C", "S72036B", "S72036C", "S72041B", "S72041C", "S72042B", "S72042C", "S72043B", "S72043C", "S72044B", "S72044C", "S72045B", "S72045C", "S72046B", "S72046C", "S72051B", "S72051C", "S72052B", "S72052C", "S72059B", "S72059C", "S72061B", "S72061C", "S72062B", "S72062C", "S72063B", "S72063C", "S72064B", "S72064C", "S72065B", "S72065C", "S72066B", "S72066C", "S72091B", "S72091C", "S72092B", "S72092C", "S72099B", "S72099C", "S72101B", "S72101C", "S7221B", "S72102C", "S72109B", "S72109C", "S72111B", "S72111C", "S72112B", "S72112C", "S72113B", "S72113C", "S72114B", "S72114C", "S72115B", "S72115C", "S72116B", "S72116C", "S72121B", "S72121C", "S72122B", "S72122C", "S72123B", "S72123C", "S72124B", "S72124C", "S72125B", "S72125C", "S72126B", "S72126C", "S72131B", "S72131C", "S72132B", "S72132C", "S72133B", "S72133C", "S72134B", "S72134C", "S72135B", "S72135C", "S72136B", "S72136C", "S72141B", "S72141C", "S72142B", "S72142C", "S72143B", "S72143C", "S72144B", "S72144C", "S72145B", "S72145C", "S72146B", "S72146C", "S7221XB", "S7221XC", "S72221XB", "S7222XC", "S7223XB", "S7223XC", "S7224XB", "S7224XC", "S7225XB", "S7225XC", "S7226XB", "S7226XC", "S72301B", "S72301C", "S72302B", "S72302C", "S72309B", "S72309C", "S72321B", "S72321C", "S72322B", "S72322C", "S72323B", "S72323C", "S72324B", "S72324C", "S72325B", "S72325C", "S72326B", "S72326C", "S72331B", "S72331C", "S72332B", "S72332C", "S72333B", "S72333C", "S72334B", "S72334C", "S72335B", "S72335C", "S72336B", "S72336C", "S72341B", "S72341C", "S72342B", "S72342C", "S72343B", "S72343C", "S72344B", "S72344C", "S72345B", "S72345C", "S72346B", "S72346C", "S72351B", "S72351C", "S72352B", "S72352C", "S72353B", "S72353C", "S72354B", "S72354C", "S72355B", "S72355C", "S72356B", "S72356C", "S72361B", "S72361C", "S72362B", "S72362C", "S72363B", "S72363C", "S72364B", "S72364C", "S72365B", "S72365C", "S72366B", "S72366C", "S72391B", "S72391C", "S72392B", "S72392C", "S72399B", "S72399C", "S72401B", "S72401C", "S72402B", "S72402C", "S72409B", "S72409C", "S72411B", "S72411C", "S72412B", "S72412C", "S72413B", "S72413C", "S72414B", "S72414C", "S72415B", "S72415C", "S72416B", "S72416C", "S72421B", "S72421C", "S72422B", "S72422C", "S72423B", "S72423C", "S72424B", "S72424C", "S72425B", "S72425C", "S72426B", "S72426C", "S72431B", "S72431C", "S72432B", "S72432C", "S72433B", "S72433C", "S72434B", "S72434C", "S72435B", "S72435C", "S72436B", "S72436C", "S72441B", "S72441C", "S72442B", "S72442C", "S72443B", "S72443C", "S72444B", "S72444C", "S72445B", "S72445C", "S72446B", "S72446C","S72451B", "S72451C", "S72452B", "S72452C",

	"S72453B", "S72453C", "S72454B", "S72454C", "S72455B", "S72455C", "S72456B", "S72456C", "S72461B", "S72461C", "S72462B", "S72462C", "S72463B", "S72463C", "S72464B", "S72464C", "S72465B", "S72465C", "S72466B", "S72466C", "S72491B", "S72491C", "S72492B", "S72492C", "S72499B", "S72499C", "S728X1B", "S728X1C", "S728X2B", "S728X2C", "S728X9B", "S728X9C", "S7290XB", "S7290XC", "S7291XB", "S7291XC", "S7292XB", "S7292XC", "S7221XA", "S7222XA", "S7223XA", "S7224XA", "S7225XA", "S7226XA", "S72301A", "S72302A", "S72309A", "S72321A", "S72322A", "S72323A", "S72324A", "S72325A", "S72326A", "S72331A", "S72332A", "S72333A", "S72334A", "S72335A", "S72336A", "S72341A", "S72342A", "S72343A", "S72344A", "S72345A", "S72346A", "S72351A", "S72352A", "S72353A", "S72354A", "S72355A", "S72356A", "S72361A", "S72362A", "S72363A", "S72364A", "S72365A", "S72366A", "S72391A", "S72392A", "S72399A", "S72401A", "S72402A", "S72409A", "S72411A", "S72412A", "S72413A", "S72414A", "S72415A", "S72416A", "S72421A", "S72422A", "S72423A", "S72424A", "S72425A", "S72426A", "S72431A", "S72432A", "S72433A", "S72434A", "S72435A", "S72436A", "S72441A", "S72442A", "S72443A", "S72444A", "S72445A", "S72446A", "S72451A", "S72452A", "S72453A", "S72454A", "S72455A", "S72456A", "S72461A", "S72462A", "S72463A", "S72464A", "S72465A", "S72466A", "S72491A", "S72492A", "S72499A", "S728X1A", "S728X2A", "S728X9A", "S7290XA", "S7291XA", "S7292XA"
Hemiarthroplasty	"0SRA009", "0SRA019", "0SRA039", "0SRA0J9", "0SRE009", "0SRE019", "0SRE039", "0SRE0J9", "0SRR019", "0SRR039", "0SRR0J9", "0SRS019", "0SRS039", "0SRS0J9", "0SRA00A", "0SRA01A", "0SRA03A", "0SRA0JA", "0SRE00A", "0SRE01A", "0SRE03A", "0SRE0JA", "0SRR01A", "0SRR03A", "0SRR0JA", "0SRS01A", "0SRS03A", "0SRS0JA", "0SRR01Z", "0SRR03Z", "0SRR07Z", "0SRR0JZ", "0SRR0KZ", "0SRS01Z", "0SRS03Z", "0SRS07Z", "0SRS0JZ", "0SRS0KZ", "0SRA00Z", "0SRA01Z", "0SRA03Z", "0SRA07Z", "0SRA0JZ", "0SRA0KZ", "0SRE00Z", "0SRE01Z", "0SRE03Z", "0SRE07Z", "0SRE0JZ", "0SRE0KZ"
Prior total or partial hip arthroplasty	"Z9664", "Z96641", "Z96642," "Z96643", "Z96649"
Revision total or partial hip arthroplasty	"0SW909Z", "0SWB09Z", "0SW908Z", "0SW938Z", "0SW948Z", "0SW9X8Z", "0SWB08Z", "0SWB38Z", "0SWB48Z", "0SWBX8Z", "0SW90BZ", "0SWB0BZ"<br><br>"0SP90EZ" **and** "0SR90EZ"<br>"0SPB0EZ" **and** "0SRB0EZ"<br>"0SP909Z" **and** "0SW909Z"<br>"0SPB09Z" **and** "0SWB09Z"

Table S2. Complication Codes

**Complication**	**ICD-10 Codes**
Dislocation of internal hip prosthesis	"T84020A", "T84021A"
Periprosthetic fracture	"M9701XA", "M9702XA"
Intraoperative pelvic fracture	"M9665"
Intraoperative femur fracture	"M96661", "M96662", "M96669"
Periprosthetic joint infection	"T8451XA", "T8452XA"

Broken internal hip prosthesis	“T84010A”, “T84011A”
Mechanical loosening of internal hip prosthetic joint	“T84030A”, “T84031A”
Periprosthetic osteolysis of internal prosthetic hip joint	“T84050A”, “T84051A”
Wear of articular bearing surface of internal prosthetic hip joint	“T84060A”, “T84061A”
Other mechanical complication of internal hip prosthesis	“T84090A”, “T84091A”
Embolism due to internal orthopedic prosthetic devices, implants and grafts	“T8481XA”
Fibrosis due to internal orthopedic prosthetic devices, implants and grafts	“T8482XA”
Hemorrhage due to internal orthopedic prosthetic devices, implants and grafts	“T8483XA”
Pain due to internal orthopedic prosthetic devices, implants and grafts	“T8484XA”
Stenosis due to internal orthopedic prosthetic devices, implants and grafts	“T8485XA”
Thrombosis due to internal orthopedic prosthetic devices, implants and grafts	“T8486XA”
Other specified complication of internal orthopedic prosthetic devices, implants and grafts	“T8489XA”
Postprocedural shock	“T8110XA”, “T8111XA”, “T8112XA”, “T8119XA”
Wound dehiscence, unspecified	“T8130XA”
Superficial wound dehiscence	“T8131XA”
Deep wound dehiscence	“T8132XA”
Disruption of traumatic injury wound repair	“T8133XA”
Infection following a procedure, unspecified	“T8140XA”
Superficial surgical site infection following a procedure	“T8141XA”
Deep surgical site infection following a procedure	“T8142XA”
Sepsis following a procedure	“T8144XA”
Infection following a procedure, other surgical site	“T8149XA”
Major osseous defect, pelvic region and thigh	“M89751”, “M89752”, “M89759”
Toxic effect of chromium and its compounds, accidental (unintentional)	“T562X1A”

Toxic effect of unspecified metal (cobalt), accidental (unintentional)	“T5691XA”
Intraoperative hemorrhage and hematoma	“M96810”
Postprocedural hemorrhage	“M96830”
Postprocedural hematoma	“M96840”
Postprocedural seroma	“M96842”
Other intraoperative and postprocedural complications	“M9689”
Acute myocardial infarction	“I210”, “I2101”, “I2102”, “I2109”, “I211”, “I2111”, “I2119”, “I212”, “I2121”, “I2129”, “I213”, “I214”, “I219”, “I21A”, “I21A1”, “I21A9”, “I220”, “I221”, “I222”, “I228”, “I229”
Pneumonia	“J120”, “J121”, “J122”, “J123”, “J128”, “J1281”, “J1289”, “J129”, “J13”, “J14”, “J150”, “J151 “J152”, “J1520”, “J1521”, “J15211”, “J15212”, “J1529”, “J153”, “J154”, “J155”, “J156”, “J157”, “J158”, “J159”, “J160”, “J168”, “J17”, “J180”, “J181”, “J182”, “J188”, “J189”
Respiratory failure	“J9600”, “J9601”, “J9602”, “J9610”, “J9611”, “J9612”, “J9620”, “J9621”, “J9622”, “J9690”, “J9691”, “J9692”, “J952”, “J953”, “J95821”, “J95822”, “J80”, “R0600”, “R0601”, “R0602”, “R0603”, “R0609”, “R069”
Acute renal failure	“N170”, “N171”, “N172”, “N178”, “N179”, “N19”
Cerebral infarction	“I6300”, “I6301”, “I63011”, “I63012”, “I63013”, “I63019”, “I6302”, “I63031”, “I63032”, “I63033”, “I63039”, “I6309”, “I6310”, “I6311”, “I63111”, “I63112”, “I63113”, “I63119”, “I6312”, “I63131”, “I63132”, “I63133”, “I63139”, “I6319”, “I6320”, “I6321”, “I63211”, “I63212”, “I63213”, “I63219”, “I6322”, “I63231”, “I63232”, “I63233”, “I63239”, “I6329”, “I6330”, “I63311”, “I63312”, “I63313”, “I63319”, “I63321”,“I63322”,“I63323”, “I63329”, “I63331”,“I63332”,“I63333”, “I63339”, “I63341”,“I63342”,“I63343”, “I63349”, “I6339”, “I6340”, “I63411”, “I63412”, “I63413”, “I63419”, “I63421”,“I63422”,“I63423”, “I63429”, “I63431”,“I63432”,“I63433”, “I63439”, “I63441”,“I63442”,“I63443”, “I63449”, “I6349”,“I6350”, “I63511”, “I63512”, “I63513”, “I63519”, “I63521”,“I63522”,“I63523”, “I63529”, “I63531”,“I63532”,“I63533”, “I63539”, “I63541”,“I63542”,“I63543”, “I63549”, “I6359”, “I636”, “I6381”, “I6389”, “I639”
Peripheral nerve injury	“S7400XA”, “S7402XA”, “S7402XA”, “S7410XA”, “S7411XA”, “S7412XA”, “S8400XA”, “S8401XA”, “S8402XA”, “S8410XA”, “S8411XA”, “S8412XA”, “S7420XA”, “S7421XA”, “S7422XA”, “S8420XA”, “S8421XA”, “S8422XA”, “S748X1A”, “S748X2A”, “S748X9A”, “S7490XA”, “S7491XA”, “S7492XA”, “S84801A”, “S84802A”, “S84809A”, “S8490XA”, “S8491XA”, “S8492XA”, “G5700”, “G5701”, “G5702”, “G5703”, “G5710”, “G5711”, “G5712”, “G5713”, “G5720”, “G5721”, “G5722”, “G5723”, “G5730”, “G5731”, “G5732”, “G5733”, “G5740”, “G5741”, “G5742”, “G5743”, “G5770”, “G5771”, “G5772”, “G5773”, “G5780”, “G5781”, “G5782”, “G5783”, “G5790”, “G5791”, “G5792”, “G5793”
Postoperative ileus	“K567”
Urinary tract infection	“N390”
